# Oncogenic role and potential regulatory mechanism of topoisomerase IIα in a pan-cancer analysis

**DOI:** 10.1038/s41598-022-15205-7

**Published:** 2022-07-01

**Authors:** Xiaobo Wang, Jinhua Wang, Li Lyu, Xin Gao, Yinuo Cai, Bo Tang

**Affiliations:** 1 Department of Hematology, The Second Affiliated Hospital of Dalian Medical University, Liaoning Dalian, People’s Republic of China; 2 Department of Pathology, The Second Affiliated Hospital of Dalian Medical University, Dalian, Liaoning People’s Republic of China

**Keywords:** Tumour immunology, Oncogenes

## Abstract

Topoisomerase IIα (TOP2A) plays an oncogenic role in multiple tumor types. However, no pan-cancer analysis about the function and the upstream molecular mechanism of TOP2A is available. For the first time, we analyzed potential oncogenic roles of TOP2A in 33 cancer types via The Cancer Genome Atlas (TCGA) database. Overexpression of TOP2A was existed in almost all cancer types, and related to poor prognosis and advanced pathological stages in most cases. Besides, the high frequency of TOP2A genetic alterations was observed in several cancer types, and related to prognosis in some cases. Moreover, we conduct upstream miRNAs and lncRNAs of TOP2A to establish ceRNA networks in kidney renal clear cell carcinoma (SNHG3-miR-139-5p), kidney renal papillary cell carcinoma (TMEM147-AS1/N4BP2L2-IT2/THUMPD3-AS1/ERICD/TTN-AS1/SH3BP5-AS1/THRB-IT1/SNHG3/NEAT1-miR-139-5p), liver hepatocellular carcinoma (SNHG3/THUMPD3-AS1/NUTM2B-AS1/NUTM2A-AS1-miR-139-5p and SNHG6/GSEC/SNHG1/SNHG14/LINC00265/MIR3142HG-miR-101-3p) and lung adenocarcinoma (TYMSOS/HELLPAR/SNHG1/GSEC/SNHG6-miR-101-3p). TOP2A expression was generally positively correlated with cancer associated fibroblasts, M0 and M1 macrophages in most cancer types. Furthermore, TOP2A was positively associated with expression of immune checkpoints (CD274, CTLA4, HAVCR2, LAG3, PDCD1 and TIGIT) in most cancer types. Our first TOP2A pan-cancer study contributes to understanding the prognostic roles, immunological roles and potential upstream molecular mechanism of TOP2A in different cancers.

## Introduction

Functioning as a factor of DNA unlinking, topoisomerase IIα (TOP2A) not only exerts a key role in cell proliferation, DNA replication, transcription and recombination, but also participates in other biological processes such as chromosome concentration and separation^1^. TOP2A expression is considered as cell cycle-dependent and peaks in the G2/M phase^2^. TOP2A overexpression associates with the outcomes of patients in multiple tumor types, suggesting its potential carcinogenic effect and prognostic value^3–8^. Previous studies have respectively reported partial molecular mechanism of TOP2A in several tumor types. For example, TOP2A functions as an oncogenic factor in the tumorigenesis and development by regulating various pathways including AKT and ERK pathway in colon cancer, β-catenin pathway in pancreatic cancer as well as MAPK pathway in lung adenocarcinoma (LUAD)^7,9,10^. Besides, it has been reported that TOP2A participates in the regulation of tumor progression by interacting with other genes such as MDM4 and CENPF^11,12^.

Tumor microenvironment (TMA) including immune cells and tumor stromal cells also makes influence in tumor occurrence and progression. Effector T cells, dendritic cells (DCs), M1 macrophages and natural killer cells function as antineoplastic factors, while cancer associated fibroblasts (CAFs) and M2 macrophages act as tumor-promoting factors in the processes of tumor proliferation, tumor invasion, and tumor metastasis^13,14^. Besides, immune suppressor cells including regulatory T cells (Tregs) play a part in the disruption of immune surveillance by inhibiting the proliferation of B and T cells as well as disrupting DCs’ antigen presentation^13^. Meanwhile, immune escape via immune checkpoints has also been viewed as a vital mechanism in tumor occurrence and progression. Immunotherapy targeting immune checkpoint is a hot spot in current clinical research and it is considered as one of effective means in antitumor therapy. Programmed cell death ligand 1 (PD-L1/CD274), cytotoxic T lymphocyte-associated antigen 4 (CTLA-4) and programmed cell death 1 (PD-1/PDCD1) are three frequently targeted immune checkpoints, but patients’ response rates are still limited. Hence, more targets need to be explored for the expansion of therapeutic range and improvement of existing response rates. Three immune checkpoints, namely T cell immunoglobulin domain and mucin domain 3 (TIM-3/HAVCR2), lymphocyte activation gene 3 (LAG-3) as well as T cell immunoreceptor with Ig and ITIM domains (TIGIT), are being under clinical trials. LAG-3 negatively regulates the activation of T cells and synergizes with PDCD1 in the mediation of T cell exhaustion^15^. Meanwhile, HAVCR2 has a certain association with T cell exhaustion, and TIGIT plays an important role in the limitation of T cell inflammation^16,17^.

A competing endogenous RNA (ceRNA) hypothesis described that lncRNAs, mRNAs, and transcribed pseudogenes compete binding miRNAs via acting as natural miRNA sponges by virtue of sharing no less than one miRNA response element^18^. The ceRNA network targeting TOP2A has significant effects on tumor occurrence and development in several tumor types. For instance, it has been reported that TOP2A is modulated by zinc finger protein 148 via miR-101, miR-144, miR-335 as well as miR-365 in the cell proliferation of colorectal cancer^19^. In terms of gastric cancer, FAM230B has been confirmed to increase TOP2A expression via miR-27a-5p in tumor development and metastasis^20^. Although there have been numerous studies focusing on TOP2A’s oncogenic role and regulatory networks in tumors, we found that there was no comprehensive systematic analysis of TOP2A from the pan-cancer aspect available. Meanwhile, more ceRNA regulatory networks need to be explored for in-depth understanding of TOP2A’s molecular mechanism in multiple tumor types. To supplement the existing molecular mechanisms, we predicted the upstream miRNAs of TOP2A and corresponding lncRNAs, and investigated underlying ceRNA networks. In consideration of TMA’s important role in tumors, we explored the potential relationship between TOP2A and immune infiltration. Moreover, we also investigated the correlation between TOP2A and above six immune checkpoints to provide more potential options for tumor therapy.

Taken together, we comprehensively conduct a pan-cancer analysis about TOP2A based on the clinical data of The Cancer Genome Atlas (TCGA) database for the first time, including gene expression, clinical survival prognosis, pathological stage, genetic alteration, immune cell infiltration, and immune checkpoints. Moreover, we conduct upstream miRNAs and lncRNAs of TOP2A to establish the ceRNA network in kidney renal clear cell carcinoma (KIRC), kidney renal papillary cell carcinoma (KIRP), liver hepatocellular carcinoma (LIHC) and LUAD by expression analysis, survival analysis, and correlation analysis. We believe that our work could assist in perfecting existing molecular and immunological mechanisms of TOP2A, exploring TOP2A’s prognostic value as well as providing more possibilities for antitumor therapy.

## Methods

### Data source and processing

The Cancer Genome Atlas (TCGA; http://cancergenome.nih.gov) is a landmark cancer genomics program, and characterized over 20,000 primary cancer and normal samples in 33 cancer types until Oct, 2021. We collected gene expression RNAseq data and survival data from multiple cancer and normal samples of TCGA database by UCSC Xena (https://xenabrowser.net/)^21^. Genotype-tissue expression (GTEx; http://commonfund.nih.gov/GTEx/) is a gene expression data from 54 normal tissue sites across nearly 1000 people by RNA sequencing. We used normal samples from GTEx when there were not enough (less than 5) normal samples from TCGA in specific cancer types, to compare TOP2A expression from cancer and normal tissue.

### TOP2A expression profiles

Perl script and R language were used to organize the data. R language was used to analyze TOP2A expression in cancer types with enough (more than 5) normal tissues from TCGA project. We take |log2FC| > 1 and an adjusted p-value < 0.05 as the cut-off criterion for further TOP2A-ceRNA network analysis. For certain tumor types without enough normal tissues from TCGA project, we used the “Expression on Box Plots” module of the GEPIA2 (Gene Expression Profiling Interactive Analysis) web server (http://gepia2.cancer-pku.cn) to obtain TOP2A expression in these tumor tissues and the normal tissues of the GTEx and TCGA database^22^.

### Survival analysis

We analyzed overall survival (OS) and disease-free survival (DFS) based on TOP2A expression (50% high-expression group and 50% low-expression group) in different cancer types by the “Survival Plots” module of the GEPIA2 web server. GEPIA uses Log-rank test, also known as the Mantel–Cox test, for hypothesis test. Furthermore, we performed OS analysis using “survival” package in R by the Kaplan–Meier survival curve and Log-rank test based on TOP2A expression (50% high-expression group and 50% low-expression group) in cancer types with enough (more than 5) TCGA adjacent normal tissues, and only the tumor types with statistical significance by these two methods will undergo further TOP2A -ceRNA network analysis. We also performed survival analysis by Kaplan–Meier survival curve based on miRNA and lncRNA expression (best cutoff), which used “survival” and “survminer” packages in R.

### Genetic alteration analysis

We used cBioPortal web (https://www.cbioportal.org/) to obtain genetic alteration characteristics of TOP2A^23^. The “Cancer Types Summary” module was used to find the alteration frequency, mutation type across all TCGA tumors types. We also obtained the overall, disease-free, progression-free, and disease-free survival differences in TCGA cancer cases with or without TOP2A genetic alteration.

### Prediction of upstream miRNA/lncRNA

The Starbase database (https://starbase.sysu.edu.cn/) was employed to predict miRNA-lncRNA interactions and miRNA-mRNA interactions^24^, and the results were supported by Ago CLIP-seq data. Interactions of miRNA-target were predicted by at least 2 programs from PITA, RNA22, miRmap, DIANA-microT, miRanda, PicTar and TargetScan. The interactions of miRNA-lncRNA were predicted by using miRanda program.

### Immune infiltration

TIMER2.0 (http://timer.cistrome.org/) web server is a comprehensive resource for systematical analysis of immune infiltrates across diverse cancer types^25,26^. We used it to explore the associations between TOP2A expression and immune infiltrates (B cells, CD4+ T cells, CD8+ T cells, cancer associated fibroblasts, myeloid dendritic cells, macrophages, monocytes, NK cells, Tregs, and neutrophil) across TCGA tumors. Moreover, we used “gene correlation” module of TIMER2.0 for exploring the correlations between TOP2A and expressions of immune checkpoints (CD274, CTLA4, HAVCR2, LAG3, PDCD1 and TIGIT). The *p*-values and partial rho values were obtained via the purity-adjusted Spearman’s rank correlation test.

### Correlation analysis

The correlations between TOP2A expression and TOP2A -bound miRNAs, TOP2A -bound miRNAs and upstream lncRNAs, and TOP2A expression and upstream lncRNAs were analyzed by R language. The *p*-values and partial rho values were obtained by the Spearman’s rank correlation test.

## Results

### TOP2A was over-expressed in most cancers

**Figure 1:**
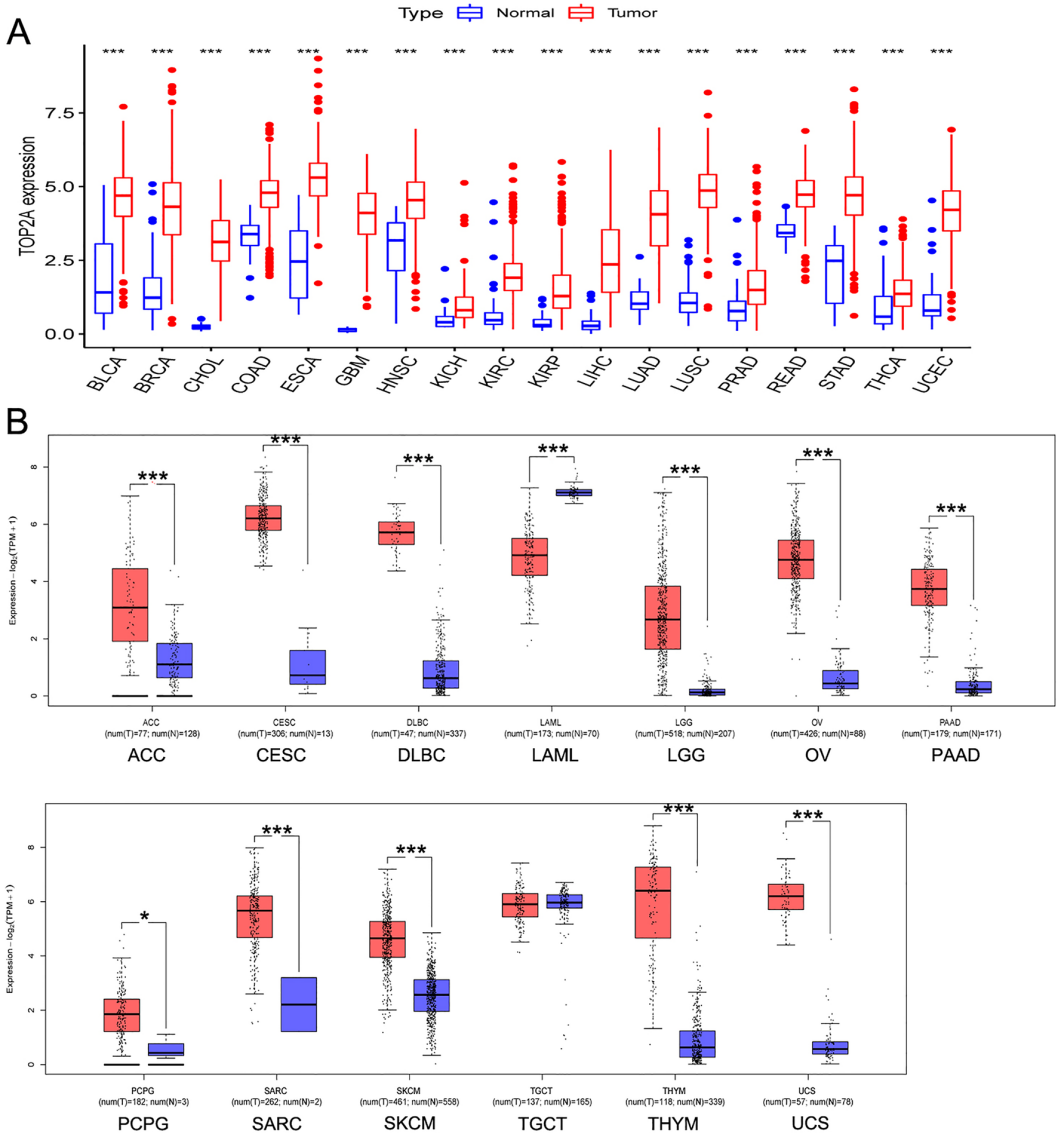
Expression analysis of TOP2A in various cancers. (**A**) The expression of the TOP2A in 18 cancer types with 5 or more normal tissues from TCGA project via R. (**B**) The expression of the TOP2A in 13 other cancer types, normal tissues from GTEx project or TCGA + GTEx as controls. *p < 0.05, ***p < 0.001.

We first analyzed TOP2A expression in cancer types with enough adjacent normal tissues in TCGA project. As graphed in Fig. 1A, TOP2A expression in all 18 cancer types of bladder urothelial carcinoma (BLCA), breast invasive carcinoma (BRCA), cholangiocarcinoma (CHOL), colon adenocarcinoma (COAD), esophageal carcinoma (ESCA), glioblastoma multiforme (GBM), head and neck squamous cell carcinoma (HNSC), kidney chromophobe (KICH), KIRC, KIRP, LIHC, LUAD, lung squamous cell carcinoma (LUSC), prostate adenocarcinoma (PRAD), rectum adenocarcinoma (READ), stomach adenocarcinoma (STAD), thyroid carcinoma (THCA), uterine corpus endometrial carcinoma (UCEC) was higher than their adjacent normal tissues (all *p* values < 0.001). Besides, we performed TOP2A expression in other cancer types which include the normal tissues from GTEx project as controls. As shown in Fig. 1B, TOP2A expression was higher in adrenocortical carcinoma (ACC), cervical squamous cell carcinoma (CESC), diffuse large B-cell lymphoma (DLBC), lower grade glioma (LGG), ovarian serous cystadenocarcinoma (OV), pancreatic adenocarcinoma (PAAD), pheochromocytoma and paraganglioma (PCPG), sarcoma (SARC), skin cutaneous melanoma (SKCM), thymoma (THYM) and uterine carcinosarcoma (UCS, *p* < 0.05 in PCPG, and all other *p* values < 0.001). However, the expression of TOP2A was lower in acute myeloid leukemia (LAML) compared with normal tissues (*p* < 0.001), and we did not obtain a significant difference in testicular germ cell tumors (TGCT).

### Significant prognostic value of TOP2A in cancers

**Figure 2:**
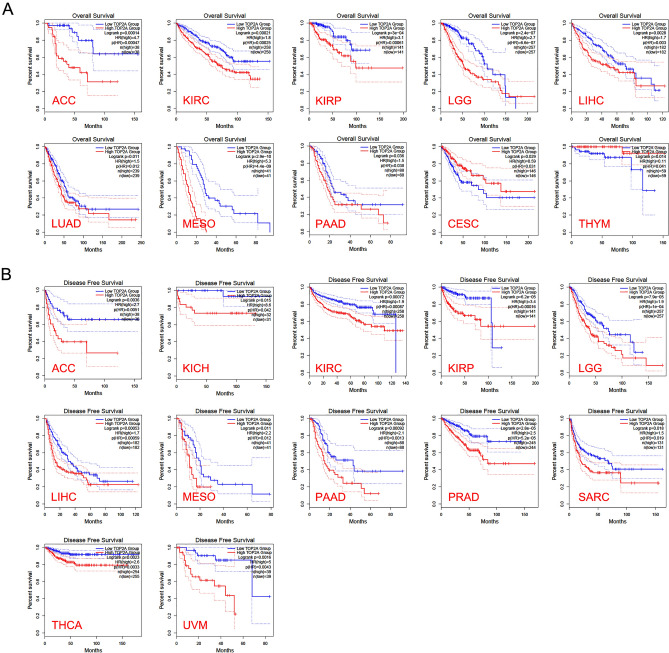
The overall survival analysis (**A**) and disease-free survival analysis (**B**) based on TOP2A expression in various cancer.

**Figure 3:**
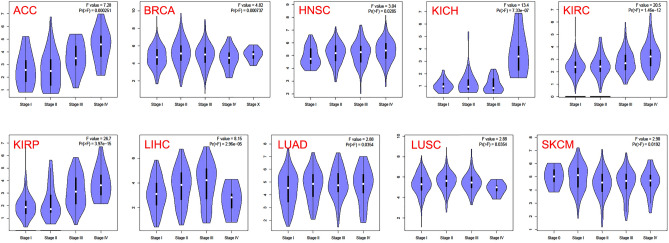
TOP2A expression of various pathological stages in various cancers.

We used the “Survival Plots” module of GEPIA2 to analyze the relationship between TOP2A expression and the clinical prognosis of patients with different cancer types in TCGA project. We separated the cancer cases into high-expression group (50%) and low-expression group (50%) according to the expression levels of TOP2A. As showed in Fig. 2A, high expression of TOP2A was related to poor overall survival (OS) in cancers of ACC (*p* < 0.001), KIRC (*p* < 0.001), KIRP (*p* < 0.001), LGG (*p* < 0.001), LIHC (*p* = 0.003), LUAD (*p* = 0.011), MESO (*p* < 0.001) and PAAD (*p* = 0.038). However, high expression of TOP2A was related to better OS in CESC (*p* = 0.031) and THYM (*p* = 0.041). Disease-free survival (DFS) data indicated high expression of TOP2A was related to poor DFS in cancers of ACC (*p* = 0.005), KICH (*p* = 0.042), KIRC (*p* < 0.001), KIRP (*p* < 0.001), LGG (*p* < 0.001), LIHC (*p* < 0.001), MESO (*p* = 0.012), PAAD (*p* = 0.001), PRAD (*p* < 0.001), SARC (*p* = 0.019), THCA (*p* = 0.003) and UVM (*p* = 0.004) (Fig. 2B). In addition, we employed the “Pathological Stage Plot” module of GEPIA2 to observe the correlation between TOP2A expression and the pathological stages of cancers. As showed in Fig. 3, TOP2A expression was related to pathological stages in ACC (*p* < 0.001), BRCA (*p* < 0.001), HNSC (*p* = 0.029), KICH (*p* < 0.001), KIRC (*p* < 0.001), KIRP (*p* < 0.001), LIHC (*p* < 0.001), LUAD (*p* = 0.035), LUSC(*p* = 0.035) and SKCM (*p* = 0.019). Generally, high expression of TOP2A was related to advanced pathological stages in most cancer types.

### Genetic alteration analysis of TOP2A

**Figure 4:**
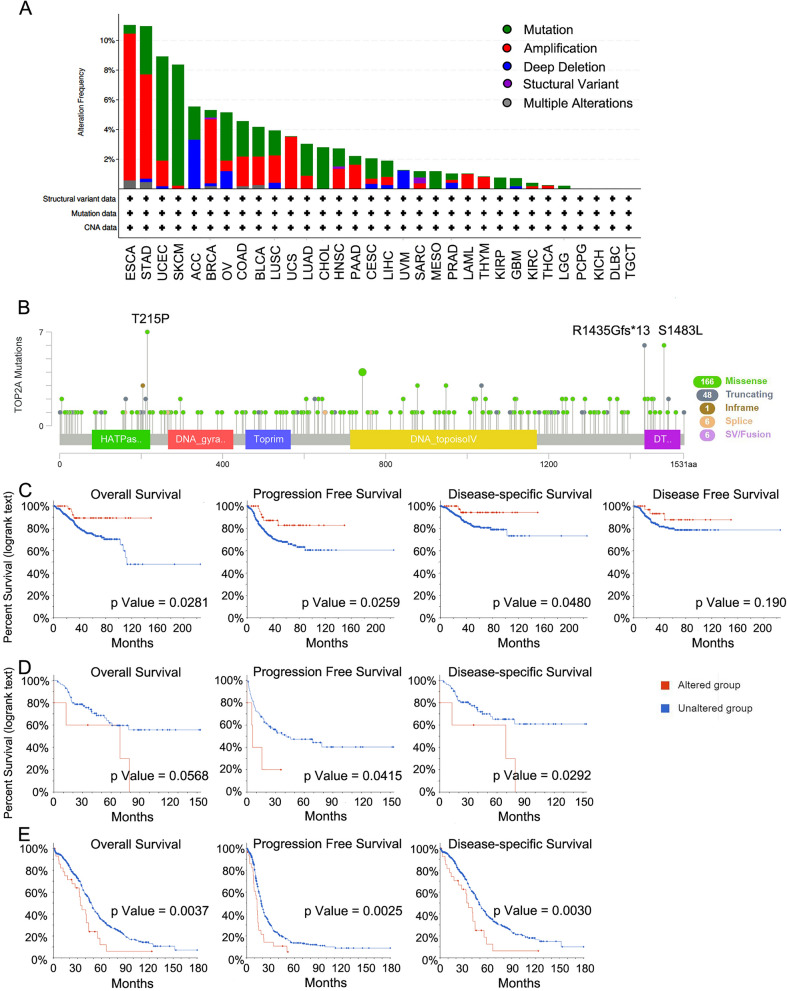
Genetic alteration analysis of TOP2A. (**A**,**B**) The alteration frequency with mutation type (**A**) and mutation site (**B**) in a pan-cancer analysis. (**C**) The correlation between TOP2A mutation status and overall, disease-specific, disease-free and progression-free survival in UCEC. (**D**) The correlation between TOP2A mutation status and overall, disease-specific, and progression-free survival in ACC. (**E**) The correlation between TOP2A mutation status and overall, disease-specific, and progression-free survival in OV.

We further investigated TOP2A genetic alteration information through the online database cBioPortal in various tumor tissues dominated from TCGA datasets (Fig. 4A). Genetic alterations in TOP2A were dominated by amplification and mutation types, which differs in different cancer types. However, deep deletion was the primary type in ACC and UVM. The high frequency of TOP2A alterations was observed in ESCA (10.99%, mainly amplification), STAD (10.91%, mainly amplification), UCEC (8.88%, mainly mutation), SKCM (8.33%, mainly mutation), ACC (5.49%, mainly deep deletion), BRCA (5.26%, mainly amplification) and OV (5.14%, mainly mutation). Figure 4B showed the types, sites and case numbers of TOP2A genetic mutations across different cancer types. The T215P missense mutation was the most common type, and it was detected in 7 OV cases. The R1435Gfs*13 truncating mutation was found in 5 STAD cases and 1 UCEC case. The S1483L missense mutation was detected in 3 UCRC cases, 1 BLCA case, 1 GBM case, and 1 LGG case. Sequentially, we explored the potential association between genetic alteration of TOP2A and clinical prognosis in different cancer types. The UCEC patients with TOP2A alterations had better OS (*p* = 0.028), progression-free survival (PFS, *p* = 0.026), and disease-specific survival (*p* = 0.048) than those with unaltered TOP2A, but not in DFS (*p* = 0.190, Fig. 4C). However, ACC patients with TOP2A alterations had poorer PFS (*p* = 0.042), and disease-specific survival (*p* = 0.029), than those with unaltered TOP2A, and OS nearly reached statistically significant (*p* = 0.057, Fig. 4D). In OV patients, we observed similar poorer survival in OS (*p* = 0.007), PFS (*p* = 0.002), and disease-specific survival (*p* = 0.003) in altered TOP2A patients compared to unaltered TOP2A patients (Fig. 4E).

### Predictive analysis of upstream miRNA of TOP2A

**Figure 5:**
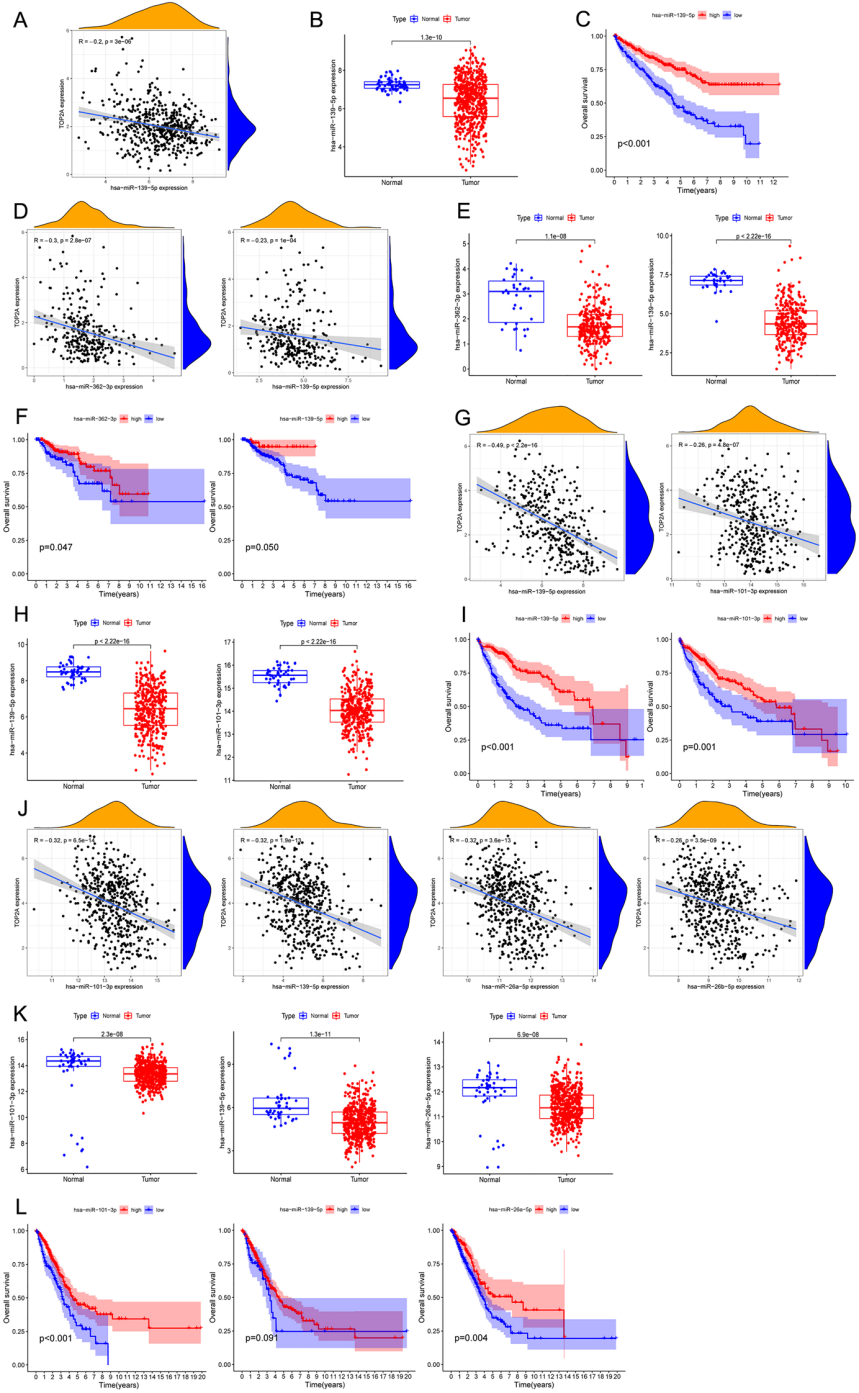
Predictive analysis of upstream miRNA of TOP2A. All miRNA expression was shown as log2(miRNA + 1). (**A**) The negative correlation between miR-139-5p and TOP2A in KIRC. (**B**) The expression of miR-139-5p in KIRC and normal tissues. (**C**) Overall survival analysis based on miR-139-5p expression in KIRC. (**D**)The negative correlation between miRNAs (miR-362-3p and miR-139-5p) and TOP2A in KIRP. (**E**) The expression of miRNAs (miR-362-3p and miR-139-5p) in KIRP and normal tissues. (**F**) Overall survival analysis based on expression of miRNAs (miR-362-3p and miR-139-5p) in KIRP. (**G**) The negative correlation between miRNAs (miR-139-5p and miR-101-3p) and TOP2A in LIHC. (**H**) The expression of miRNAs (miR-139-5p and miR-101-3p) in LIHC and normal tissues. (**I**) Overall survival analysis based on expression of miRNAs (miR-139-5p and miR-101-3p) in LIHC. (**J**) The negative correlation between miRNAs (miR-101-3p, miR-139-5p, miR-26a-5p, miR-26b-5p) and TOP2A in LUAD. (**K**) The expression of miRNAs (miR-101-3p, miR-139-5p and miR-26a-5p) in LUAD and normal tissues. (**L**) Overall survival analysis based on expression of miRNAs miR-101-3p, miR-139-5p and miR-26a-5p) in LUAD.

We further conducted predictive ceRNA networks in cancer types with enough adjacent normal tissues of TCGA project. We chose the cancer types in which TOP2A was associated with poor OS by Log-rank test and the Kaplan–Meier survival curve simultaneously (Table 1). The starBase database was used for predicting upstream miRNAs of TOP2A, and these 38 TOP2A-bound miRNAs have been supported by CLIP-Seq experiment (Supplementary Table S1). MicroRNA-139-5p was significantly negatively correlated with TOP2A expression in KIRC (R = − 0.20, *p* < 0.001, Fig. 5A), and its expression was lower in KIRC tissues compared with normal tissues (*p* < 0.001, Fig. 5B). Meanwhile, KIRC patients with low miR-139-5p expression showed poorer OS compared to the KIRC patients with high miR-139-5p expression *(p* < 0.001, Fig. 5C). In KIRP, the expression of miR-362-3p (R = − 0.30) and miR-139-5p (R = − 0.23) was negatively correlated with TOP2A (all *p* values < 0.001, Fig. 5D), and their expression was lower in KIRP tissues compared with normal tissues (all *p* values < 0.001, Fig. 5E). The KIRP patients with decreased expression levels of miR-362-3p (*p* = 0.047) and miR-139-5p (*p* = 0.05) was correlated with poor OS (Fig. 5F). In LIHC, the expression of miR-139-5p (R = − 0.49) and miR-101-3p (R = − 0.26) was negatively correlated with TOP2A (all *p* values < 0.001, Fig. 5G), and their expression was lower in LIHC tissues compared with normal tissues (all *p* values < 0.001, Fig. 5H). The LIHC patients with decreased expression levels of miR-139-5p (*p* < 0.001) and miR-101-3p (*p* = 0.001) were correlated with poor OS (Fig. 5I). In LUAD, there were four miRNAs (miR-101-3p, R = − 0.32; miR-139-5p, R = − 0.32; miR-26a-5p, R = − 0.32; miR-26b-5p, R = − 0.26) negatively correlated with TOP2A expression (all *p* values < 0.001, Fig. 5J). However, only the expressions of miR-101-3p, miR-139-5p and miR-26a-5p were lower in LUAD tissues compared with normal tissues (all *p* values < 0.001, Fig. 5K). The OS of LUAD patients with decreased expression levels of miR-101-3p (*p* < 0.001) and miR-26a-5p (*p* = 0.001) was corelated with poor prognosis (Fig. 5L). MicroRNA-139-5p had a tendency but not reached statistical significance (*p* = 0.091, Fig. 5L).

**Table 1:** TOP2A associated with poor OS by the Kaplan–Meier survival curve and Log-rank test.

CANCER TYPE	KM	HR	HR.95L	HR.95H	Cox *P* value
KIRC	0.0188866	1.6848699	1.3991101	2.0289943	3.76E−08
KIRP	1.89E-05	2.3592177	1.9020668	2.926242	5.70E−15
LIHC	0.0008658	1.2776044	1.1240224	1.4521712	0.0001774
LUAD	0.037508	1.146402	1.027697	1.278818	0.014292

### Predictive analysis of upstream lncRNAs of TOP2A-bound miRNAs

**Figure 6:**
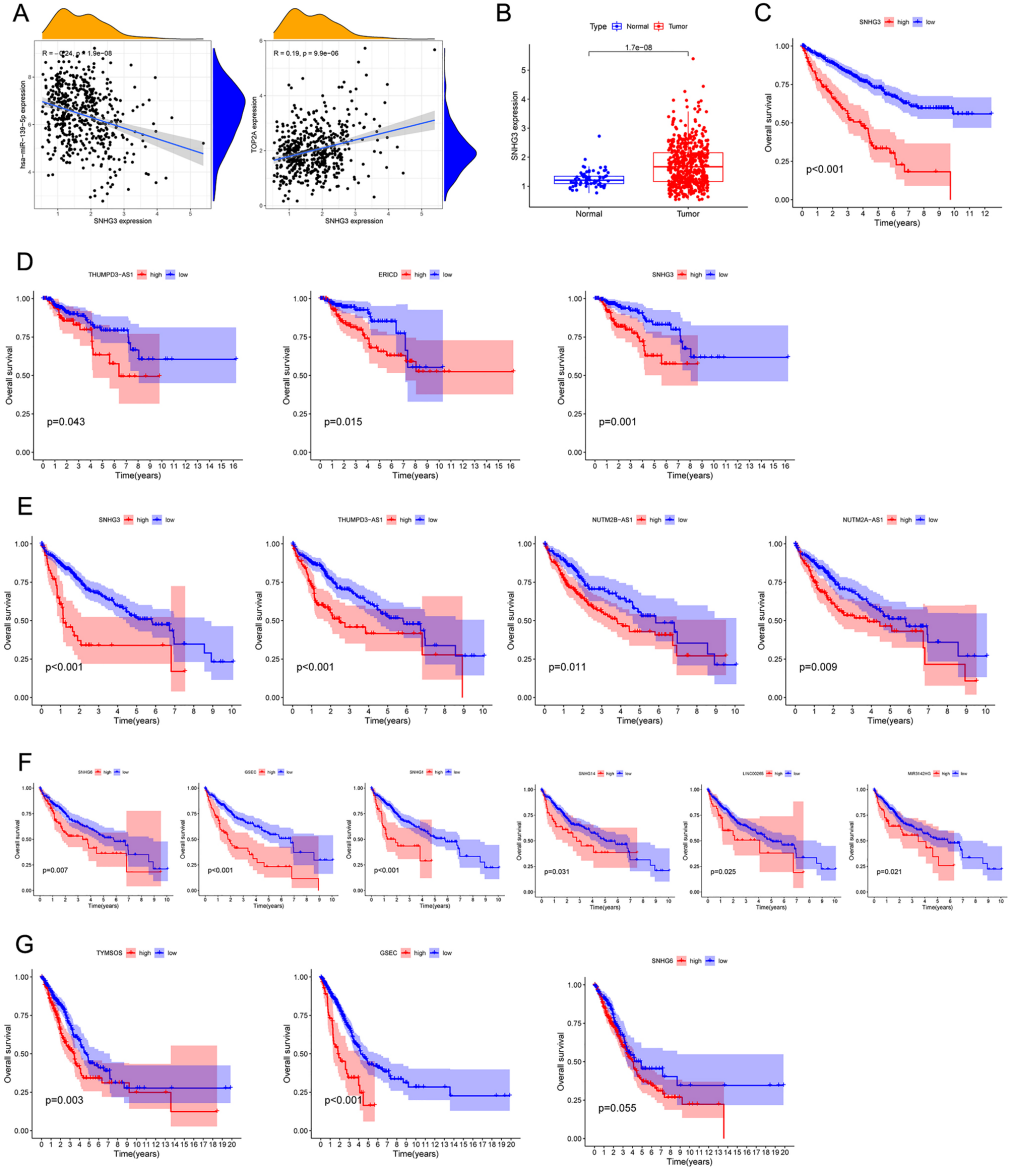
Predictive analysis of upstream lncRNAs of TOP2A -bound miRNAs. All miRNA expression was shown as log_2_(miRNA + 1). (**A**) Correlation of SNHG3 expression with miR-139-5p (left) and TOP2A (right) in KIRC. (**B**) The expression of SNHG3 in KIRC and normal tissues. (**C**) Overall survival analysis based on SNHG3 expression in KIRC. (**D**) Overall survival analysis based on miR-139-5p-bound lncRNAs (THUMPD3-AS1, ERICD and SNHG3) expression in KIRP. (**E**) Overall survival analysis based on miR-139-5p-bound lncRNAs (SNHG3, THUMPD3-AS1, NUTM2B-AS1 and NUTM2A-AS1) expression in LIHC. (**F**) Overall survival analysis based on miR-101-3p-bound lncRNAs (SNHG6, GSEC, SNHG1, SNHG14, LINC00265, MIR3142HG) expression in LIHC. (**G**) Overall survival analysis based on miR-101-3p-bound lncRNAs (TYMSOS and GSEC, and SNHG6) expression in LUAD.

The starBase database was used to predict upstream lncRNAs of the above-mentioned miRNAs with expression and survival significance in KIRC, KIRP, LIHC and LUAD. According to ceRNA mechanism, the upstream lncRNAs should be negatively correlated with target miRNAs. Moreover, the expression of lncRNAs should be higher than normal tissues. In KIRC, Small Nucleolar RNA Host Gene 3 (SNHG3) was significantly negatively correlated with miR-139-5p (R = − 0.24, *p* < 0.001), and it was positively correlated with TOP2A (R = 0.19, *p* < 0.001, Fig. 6A). Besides, the expression of SNHG3 was higher in KIRC tissues than normal tissues (*p* < 0.001, Fig. 6B). In addition, the prognosis of KIRC patients with high SNHG3 expression showed poor prognosis (*p* < 0.001, Fig. 6C).

In KIRP, TMEM147 Antisense RNA 1 (TMEM147-AS1, R = − 0.44), N4BPL2 Intronic Transcript 2 (N4BP2L2-IT2, R = − 0.39), THUMPD3 Antisense RNA 1 (THUMPD3-AS1, R = − 0.35), E2F1-Regulated Inhibitor of Cell Death (ERICD, R = − 0.34), TTN Antisense RNA 1 (TTN-AS1, R = − 0.33), SH3BP5 Antisense RNA 1 (SH3BP5-AS1, R = − 0.26), THRB Intronic Transcript 1 (THRB-IT1, R = − 0.21), Small Nucleolar RNA Host Gene 3 (SNHG3, R = − 0.20) and Nuclear Paraspeckle Assembly Transcript 1 (NEAT1, R = − 0.20) were negatively correlated with miR-139-5p (all *p* values < 0.001), and all of them were higher in KIRP tissues compared to normal tissues (all *p* values < 0.05, Table 2). The expression of ERICD (R = 0.20), SNHG3 (R = 0.16) and N4BP2L2-IT2 (R = 0.13) was positively correlated with TOP2A (all *p* values < 0.05, Table 2). Furthermore, as showed in Fig. 6D, increased expression of THUMPD3-AS1 (*p* = 0.043), ERICD (*p* = 0.015) and SNHG3 (*p* = 0.001) was significantly related to a poor OS. As for miR-362-3p, there was no lncRNA significantly negatively correlated with it in KIRP (R < − 0.20).

**Table 2:** The relationship between lncRNAs and miRNA-139-5p, lncRNAs and TOP2A, and the lncRnAs expression in KIRP and control tissues.

lncRNA	miRNA	cor	*P* value	gene	cor	*P* value	logFC	Diff *P* value
TMEM147-AS1	hsa-miR-139-5p	− 0.44031	0	TOP2A	0.079899	0.175452	0.292492	0.004841
N4BP2L2-IT2	hsa-miR-139-5p	− 0.38507	1.68E−11	TOP2A	0.133695	0.023078	0.244818	3.21E−05
THUMPD3-AS1	hsa-miR-139-5p	− 0.35231	9.38E−10	TOP2A	0.052138	0.376958	0.205441	0.036194
ERICD	hsa-miR-139-5p	− 0.33847	4.45E−09	TOP2A	0.20187	0.000569	0.425237	1.33E−09
TTN-AS1	hsa-miR-139-5p	− 0.32844	1.31E−08	TOP2A	0.093571	0.112413	0.125061	8.16E−05
SH3BP5-AS1	hsa-miR-139-5p	− 0.2645	5.58E−06	TOP2A	− 0.00528	0.928775	0.286017	0.037462
THRB-IT1	hsa-miR-139-5p	− 0.20693	0.000399	TOP2A	− 0.02023	0.732058	0.010988	0.048489
SNHG3	hsa-miR-139-5p	− 0.20287	0.000533	TOP2A	0.162037	0.005811	0.34984	0.002444
NEAT1	hsa-miR-139-5p	− 0.20181	0.000571	TOP2A	0.010542	0.858296	0.690916	0.002256

In LIHC, SNHG3 (R = − 0.43), THUMPD3-AS1 (R = − 0.38), NUTM2B Antisense RNA 1 (NUTM2B-AS1, R = − 0.29) and NUTM2A Antisense RNA 1 (NUTM2A-AS1, R = − 0.25) were significantly negatively correlated with miR-139-5p (all *p* values < 0.001), and all of them were higher in LIHC tissues compared to normal tissues (all *p* values < 0.001, Table 3). All of the above lncRNAs were positively correlated with TOP2A in LIHC (all *p* values < 0.001, Table 3). As showed in Fig. 6E, increased expression of SNHG3 (*p* < 0.001), THUMPD3-AS1 (*p* < 0.001), NUTM2B-AS1 (*p* = 0.011) and NUTM2A-AS1 (*p* = 0.009) in LIHC was significantly related to a poor OS. As for miR-101-3p, Small Nucleolar RNA Host Gene 6 (SNHG6, R = − 0.38), G-Quadruplex Forming Sequence Containing LncRNA (GSEC, R = − 0.38), Small Nucleolar RNA Host Gene 1 (SNHG1, R = − 0.35), Small Nucleolar RNA Host Gene 14 (SNHG14, R = − 0.27), Long Intergenic Non-Protein Coding RNA 265 (LINC00265, R = − 0.27) and MIR3142 Host Gene (MIR3142HG, R = − 0.25) were significantly negatively correlated with it (all *p* values < 0.001), and all of them were higher in LIHC tissues compared with normal tissues (all *p* values < 0.01, Table 3). Besides, the above miR-101-3p-bound LncRNAs were positively correlated with TOP2A (all *p* values < 0.001, Table 3). Moreover, high-expression groups of these 6 lncRNAs showed worse OS in LIHC compared to the low-expression gourps (Fig. 6F).

**Table 3:** The relationship between lncRNAs and miRNAs, lncRNAs and TOP2A, and the lncRNAs expression in LIHC and control tissues.

lncRNA	miRNA	cor	*P* value	Gene	cor	*P* value	logFC	Diff *P* value
SNHG3	hsa-miR-139-5p	− 0.42791	0	TOP2A	0.422516	0	1.077148	3.52E−23
THUMPD3-AS1	hsa-miR-139-5p	− 0.38399	2.16E−14	TOP2A	0.464942	0	0.698875	6.85E−25
NUTM2B-AS1	hsa-miR-139-5p	− 0.2915	1.30E−08	TOP2A	0.352008	4.26E−12	0.17151	1.07E−10
NUTM2A-AS1	hsa-miR-139-5p	− 0.24944	1.28E−06	TOP2A	0.326042	1.67E−10	0.324726	7.70E−14
SNHG6	hsa-miR-101-3p	− 0.38126	3.63E−14	TOP2A	0.193604	0.000184	1.451473	3.16E−21
GSEC	hsa-miR-101-3p	− 0.37963	4.88E−14	TOP2A	0.28084	4.48E−08	0.374517	2.66E−18
SNHG1	hsa-miR-101-3p	− 0.35229	4.09E−12	TOP2A	0.595842	0	1.45769	4.47E−26
SNHG14	hsa-miR-101-3p	− 0.26897	1.67E−07	TOP2A	0.595842	0	0.296678	0.006129
LINC00265	hsa-miR-101-3p	− 0.26788	1.68E−07	TOP2A	0.502874	4.14E−25	0.16592	1.79E−06
MIR3142HG	hsa-miR-101-3p	− 0.25273	8.44E−07	TOP2A	0.298437	4.77E−09	0.137124	8.36E−05

**Figure 7:**
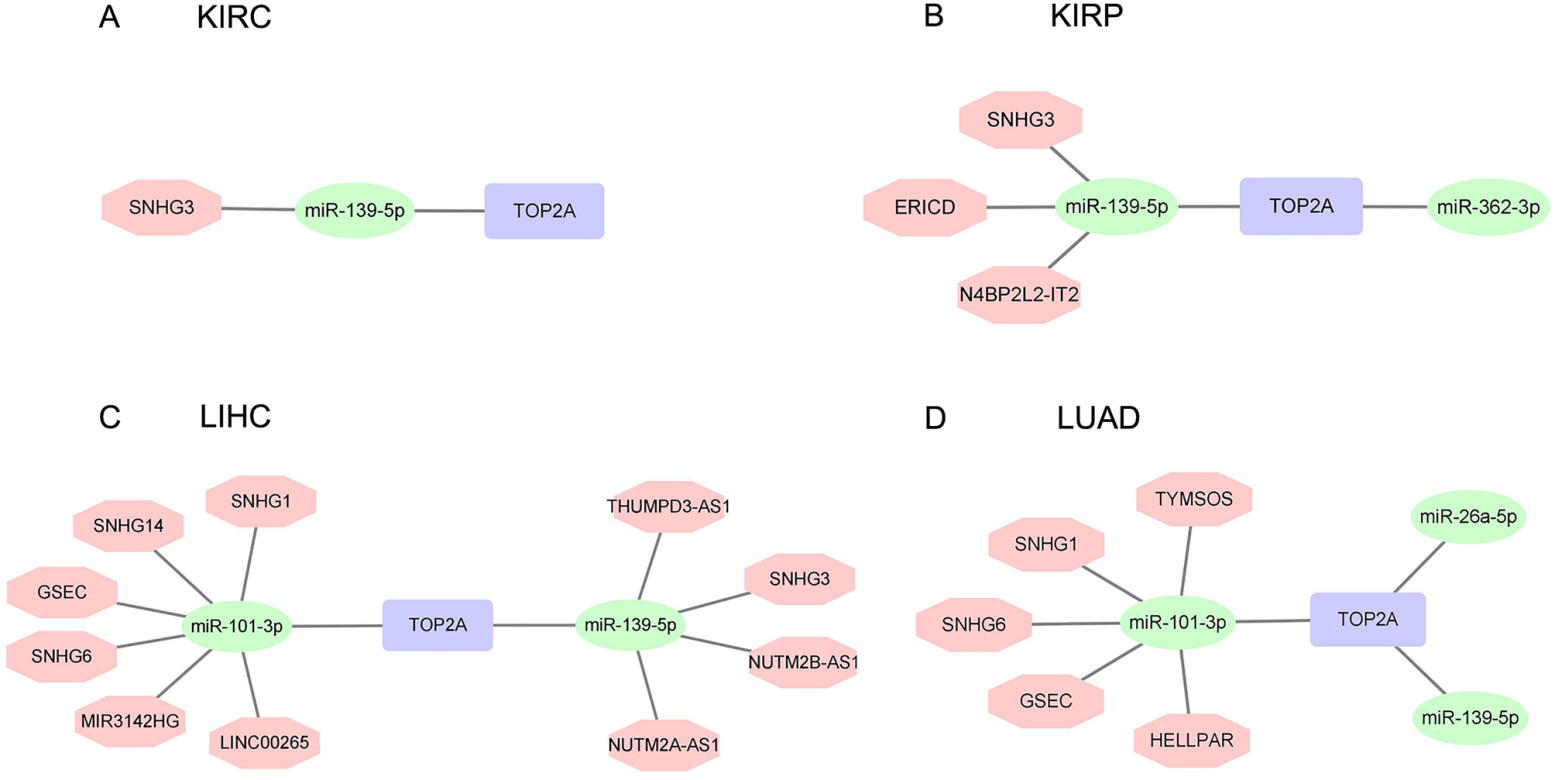
Predictive ceRNA networks of CCNA2 in KIRC KIRP, LIHC and LUAD.

In LUAD, TYMS Opposite Strand RNA (TYMSOS, R = − 0.29), HELLP Associated Long Non-Coding RNA (HELLPAR, R = − 0.26), SNHG1 (R = − 0.23), GSEC (R = − 0.22) and SNHG6 (R = − 0.21) were significantly negatively correlated with miR-101-3p (all *p* values < 0.001), and all of them were higher in LUAD tissues compared with normal tissues (all *p* values < 0.001, Table 4). In addition, these lncRNAs were positively correlated with TOP2A in LUAD (all *p* values < 0.001, Table 4). Furthermore, increased expression of TYMSOS (*p* = 0.003) and GSEC (*p* < 0.001) was significantly related to poor OS, and SNHG6 nearly reached statistically significant (*p* = 0.055, Fig. 6G). As for miR-139-5p and miR-26a-5p, there was no lncRNA significantly negatively correlated with it in LUAD (R < − 0.20). Taken together, we summarized the predictive ceRNA networks of TOP2A in KIRC, KIRP, LIHC and LUAD in Fig. 7.

**Table 4:** The lncRNAs were significantly negatively correlated with miR-101-3p in LUAD, and their expression in LUAD and control tissues.

lncRNA	miRNA	cor	*P* value	Gene	cor	*P* value	logFC	Diff *P* value
SNHG6	hsa-miR-101-3p	− 0.20543	2.91E−06	TOP2A	0.185755	2.42E−05	0.666298	1.35E−12
TYMSOS	hsa-miR-101-3p	− 0.28716	4.36E−11	TOP2A	0.468361	0	0.610323	1.01E−26
HELLPAR	hsa-miR-101-3p	− 0.26377	1.56E−09	TOP2A	0.455068	0	0.011608	8.67E−17
GSEC	hsa-miR-101-3p	− 0.21774	6.98E−07	TOP2A	0.235709	7.43E−08	0.505281	4.53E−18
SNHG1	hsa-miR-101-3p	− 0.22805	1.97E−07	TOP2A	0.346117	9.15E−16	1.309259	2.11E−27

### Correlation analysis of TOP2A with immune infiltration

Immune cells and tumor stromal cells are viewed as key components of TMA. In consideration of their certain roles in the occurrence and development of tumors, we applied TIMER approach to investigate the potential relationship between TOP2A and immune infiltration. Most algorithms showed a positive relationship between TOP2A expression and B cell infiltration in BRCA, KIRC, LIHC and PRAD, but a negative association in STAD (all *p* values < 0.05, Supplementary Fig. S1A). The infiltration level of CAFs presented a statistically positive relationship with TOP2A expression in various tumor types, namely CESE, ESCA, HNSC, HNSC-HPV−, KICH, KIRC, KIRP, LGG, PCPG, SKCM, SKCM-Metastasis as well as THCA. Contrarily, we also noticed a negative relationship between the infiltration level of CAFs and the expression of TOP2A in BRCA, LUSC, STAD and THYM (all *p* values < 0.05, Supplementary Fig. S1B). According to most algorithms, we observed a positive correlation between the infiltration level of neutrophils and TOP2A expression in the cases of BLCA, BRCA, BRCA-Basal, COAD, KICH, KIRC, KIRP, LIHC, OV as well as PAAD (all *p* values < 0.05, Supplementary Fig. S1C). The majority of algorithms suggested that infiltration level of DCs was positively correlated with the expression of TOP2A in BRCA, BRCA-Basal, KICH, LGG, LIHC, PRAD and SKCM, but negatively in STAD, TGCT and UCEC (all *p* values < 0.05, Supplementary Fig. S2A). In addition, TOP2A expression was positively related to macrophages’ infiltration level in BLCA, BRCA, KIRC, LUAD, PRAD and THCA, but negatively associated with that in BRCA-Her2, CESC, KIRP, STAD, THYM and UCEC by the most of algorithms (all *p* values < 0.05, Supplementary Fig. S2B). We further evaluated the potential association between TOP2A expression and infiltration level of different types of macrophages including M0, M1 and M2 macrophages (Supplementary Fig. S3). Based on the CIBERSORT and CIBERSORT-ABS algorithms, M0 macrophages’ infiltration level was positively related to TOP2A expression in the case of ACC, BLCA, BRCA, GBM, LIHC, LUAD, OV, SARC, STAD and THCA, but negatively in KIRP and THYM. Similarly, the infiltration level of M1 macrophages showed a positive relationship with the expression of TOP2A in BLCA, BRCA, BRCA-Basal, BRCA-LumA, BRCA-LumB, HNSC, HNSC-HPV−, HNSC-HPV+, KIRC, LGG, LIHC, LUAD, PRAD, SKCM, SKCM-Metastasis, SKCM-Primary and THCA. Contrarily, it showed a negative relationship in TGCT and THYM. Additionally, there was a positive association between the infiltration level of M2 macrophages and TOP2A expression in GBM, but a negative association in ACC, KIRP, LUAD, PAAD and STAD by the most of algorithms. As for CD8+ T-cells, most of algorithms suggested that the infiltration level was positively linked to TOP2A expression in in KIRC, LIHC, LUSC and THYM, but negatively in STAD and UCEC (all *p* values < 0.05, Supplementary Fig. S4A). Moreover, we also found that there was a positive relationship between TOP2A expression and CD4+ T-cell infiltration in HNSC, HNSC-HPV−, HNSC-HPV+, LIHC, LUSC, PRAD and THCA by both TIMER and EPIC algorithms (all *p* values < 0.05, Supplementary Fig. S4B). In terms of Tregs, the infiltration level had a positive correlation with TOP2A expression in BRCA, BRCA-LumA, KICH, KIRC, KIRP, LIHC, PCPG, PRAD and THCA, but a negative correlation in BRCA-Her2 and UCEC based on at least two algorithms (all *p* values < 0.05, Supplementary Fig. S4C).

### Correlation analysis of TOP2A with immune checkpoints

**Figure 8:**
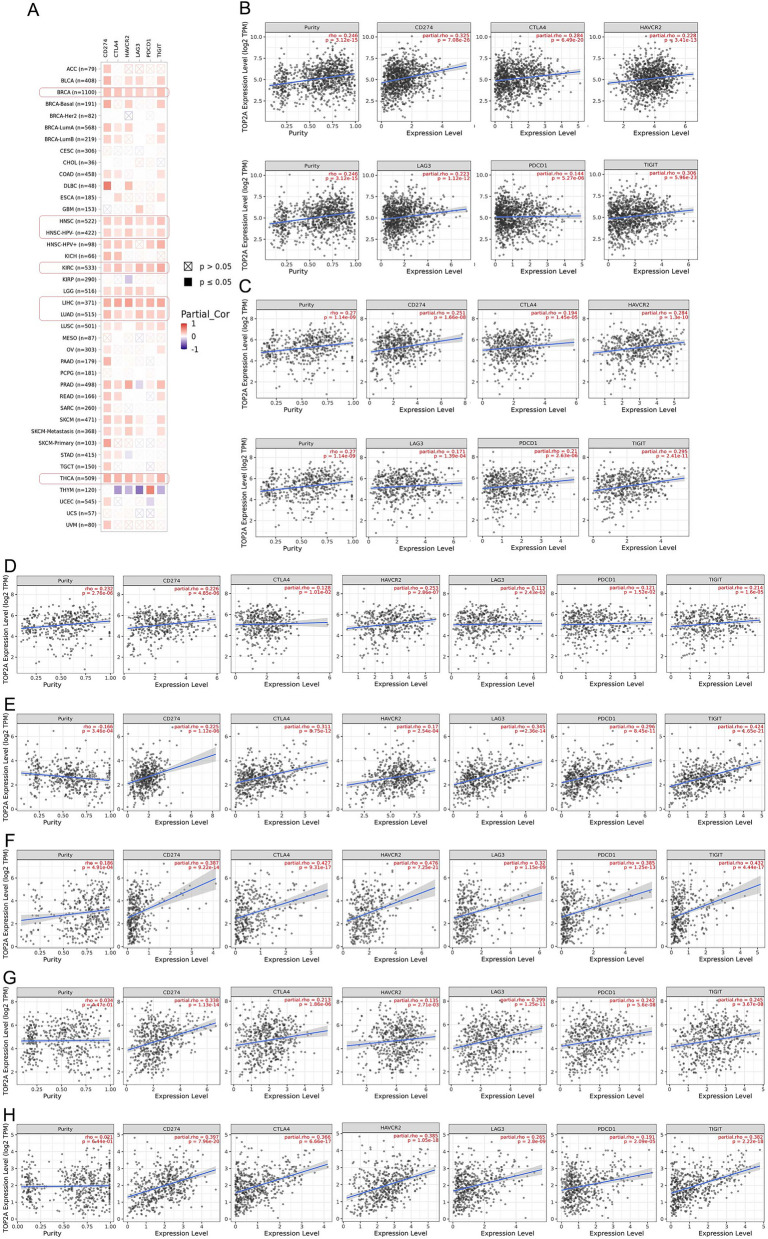
Correlation between TOP2A and immune checkpoints. (**A**) Correlation between TOP2A and immune checkpoints in multiple tumors. (**B**–**H**) Correlation between TOP2A and immune checkpoints in BRCA (**B**), HNSC (**C**), HNSC-HPV− (**D**), KIRC (**E**), LIHC (**F**), LUAD (**G**) and THCA (**H**).

To provide more possibilities of immunotherapy, we further evaluated the correlation between TOP2A expression and six immune checkpoints including CD274, CTLA-4, PDCD1, HAVCR2, LAG-3 and TIGIT (Fig. 8). The expression of CD274 was positively linked to TOP2A expression in various tumor types, including ACC, BLCA, BRCA, BRCA-Basal, BRCA-LumA, BRCA-LumB, COAD, DLBC, HNSC, HNSC-HPV−, HNSC-HPV+, KICH, KIRC, LGG, LIHC, LUAD, LUSC, OV, PAAD, PRAD, READ, SARC, SKCM, SKCM-Metastasis, SKCM-Primary, STAD, TGCT, THCA, UCEC and UVM (all *p* values < 0.05). In addition, CTLA-4 expression had a positive relationship with TOP2A expression in BRCA, BRCA-LumA, BRCA-LumB, COAD, ESCA, HNSC, HNSC-HPV−, HNSC-HPV+, KICH, KIRC, LGG, LIHC, LUAD, LUSC, PRAD, READ, SKCM, SKCM-Metastasis, STAD and THCA, but a negative relationship in THYM (all *p* values < 0.05). In terms of PDCD1, the expression was positively associated with TOP2A expression in 12 tumor types, including BRCA, HNSC, HNSC-HPV−, HNSC-HPV+, KIRC, LGG, LIHC, LUAD, LUSC, PRAD, THCA (Rho = 0.191) and THYM, but a negative relationship in UCEC (all *p* values < 0.05). Meanwhile, we also observed a positive association between HAVCR2 expression and TOP2A expression in the case of BLCA, BRCA, BRCA-Basal, BRCA-LumA, DLBC, HNSC, HNSC-HPV−, HNSC-HPV+, KIRC, LGG, LIHC, LUAD, PRAD, SKCM, SKCM-Metastasis and THCA, but a negative association in KIRP, STAD and THYM (all *p* values < 0.05). LAG-3 expression was found to have a positive relationship with TOP2A expression in BLCA, BRCA, GBM, HNSC, HNSC-HPV−, KIRC, LGG, LIHC, LUAD, LUSC, SKCM, SKCM-Metastasis and THCA, but a negative relationship in PRAD and THYM (G all *p* values < 0.05). Moreover, there was a positive correlation between TIGIT expression and TOP2A expression in BLCA, BRCA, BRCA-Basal, BRCA-LumA, BRCA-LumB, COAD, ESCA, HNSC, HNSC-HPV−, HNSC-HPV+, KIRC, LIHC, LUAD, LUSC, OV, PRAD, READ, SKCM, SKCM-Metastasis and THCA, but a negative correlation in THYM (all *p* values < 0.05).

## Discussion

As one of the important factors of DNA unlinking, TOP2A has been involved in the occurrence and development of many different cancers, including bladder urothelial carcinoma, prostate cancer, breast cancer, colon cancer and liver cancer. However, it remains unclear whether TOP2A plays a role in pathogenesis of different cancers through some common molecular mechanisms. Therefore, we performed the pan-cancer analysis of TOP2A across 33 different cancer types.

Our analysis corroborated that TOP2A was overexpressed in all 18 cancer types with enough normal tissues from TCGA project. The overexpression of TOP2A was also existed in most cancers when we added the normal tissues from GTEx program. TOP2A was only decreased in LAML. However, we found the chronic myelogenous leukemia K562 cell line was used as matched normal tissue from GTEx when compared with LAML cells. LAML cells derived from immature hematopoietic cells in bone marrow (myeloblast, promyeloytes et al.), so the immature hematopoietic cells from healthy person should be the reasonable controls. Therefore, the LAML control tissues from GTEx exist limitations. In addition, we also provide substantial evidence in support of the prognostic values of TOP2A. The Log-rank test revealed that high expression of TOP2A was a risk factor in ACC, KIRC, KIRP, LGG, LIHC, LUAD, MESO, PAAD and SCKM, and a protective factor in COAD and THYM. Part of these findings are consistent with other studies^8,27–29^, but the most of these results derived from the analysis of TCGA or GEO transcriptome data. However, some studies verified that the higher expression of TOP2A has nothing to do with the worse prognosis in these cancer types. Roca et al. showed that high expression of TOP2A has no prognostic efficacy of OS in ACC patients, but was associated with longer time to progression (TTP) after EDP-M scheme^30^. Therefore, in addition to the transcriptome data based on TCGA or GEO databases, more experiments are needed to verify the prognostic value of TOP2A in tumors.

Commonly altered at both copy number and expression level in tumor cells, TOP2A is considered as a key player of decatenation checkpoint. The alterations of TOP2A may cause defective decatenation checkpoint, and then lead to the chromosome instability and additional imbalances of chromosomes in tumor cells, which results in increased cell survival, proliferation, carcinogenesis as well as the tumors’ aggressiveness^2^. We analyzed the genetic alteration of TOP2A, and discovered that TOP2A alterations in tumors were dominated by mutation and amplification types. In terms of mutation, it has been reported that tumors with p.K743N mutation of TOP2A have potentially oncogenic indel mutations including frameshift mutations in tumor suppressors PTEN and TP53 and an activating insertion in BRAF^31^. In our study, the most common mutation type was T215P missense mutation, but there has been no report about this mutation up to date, so it can be a direction which we can explore in the future. Moreover, in most cases, gene amplification, that is the increase of gene copy number, can lead to gene overexpression which refers the increase of post-transcriptional RNA. In this study, we did demonstrate the overexpression of TOP2A in most tumor types. However, the interaction between TOP2A amplification and expression is of great complexity, and need to be further investigated for the reason that the protein overexpression is reported not always a result of TOP2A amplification^32^.

In this study, we first presented evidence of the potential ceRNA network based on TOP2A in tumors. Importantly, miRNA-139-5p was found as a common potential upstream regulator of TOP2A in KIRC, KIRP, LIHC and LUAD. As we known, there was only one report about the relationship between miRNA-139-5p and TOP2A in tumor before^33^. Although we did not use experimental verification, we conduct the relationship by expression analysis, survival analysis, and correlation analysis based on RNAseq data. Therefore, our finding is meaningful for understanding the common regulatory mechanism of TOP2A. Moreover, we found SNHG3-miR-139-5p-TOP2A network in KIRC, KIRP and LIHC. The lncRNA SNHG3 was verified as an oncogenic factor in the tumorigenesis and development by regulating miR-139-5p in various ways, including Notch pathway in ovarian cancer^34^, MYB transcription factor in gastric cancer^35^, and BMI1 protein in LIHC^36^. Importantly, Zhang et al. verified the same SNHG3-miR-139-5p-TOP2A ceRNA network by functional experiment in KIRC^33^, which demonstrate the reliability of our prediction based on transcriptome data. Besides, we found SNHG1/SNHG6/GESC-miR-101-3p-TOP2A network in LIHC and LUAD. There was no report about the regulatory relationship between miRNA-101-3p and TOP2A in tumor, but the regulatory function of SNHG1 on miR-101-3p was verified in osteosarcoma^37^. However, there exist several limitations of the prediction through those programs in the Starbase database. All of those prediction programs are based on existing knowledge about the expression and functions of non-coding RNAs (ncRNAs). Currently, numerous ncRNAs remain unknown, so existing knowledge about ncRNAs need to be continuously improved by the explorations of potential ncRNAs. Moreover, although several ncRNAs have been explored, their functions and corresponding regulatory mechanisms may differ in case of changes in expression pattern, structure as well as interacting proteins^38^. As the largest class of ncRNAs, lncRNAs are considered to have highly diverse functions and regulatory mechanisms, which increases their complexity^39^. Additionally, low abundance of most lncRNAs in cells and the tissue specificity add the difficulty of investigating the interactions between lncRNAs and proteins or nucleic acids^39,40^. Up to date, only a small subset of lncRNAs have gotten identified and functionally described in the literatures^39,41^. Therefore, the regulatory networks we discovered based on the existing Ago CLIP-seq data are required to be further confirmed.

TMA has been confirmed to have a significant effect in tumor progression and influence patients’ outcomes as well as chemotherapy drug resistance^42,43^. Thus, in-depth exploration of the potential relationship between TOP2A expression and immune infiltration is needed for the supplement of immunological mechanism and improvement of prognosis as well as therapeutic efficiency. In this study, TOP2A expression was indicated to be negatively correlated with CD8+ T-cell infiltration in UCEC. On the one hand, Tregs’ infiltration was confirmed in this study to be positively associated with TOP2A expression in UCEC. Considering Tregs’ role in the inhibition of T cell proliferation, we speculated that TOP2A expression may influence the regulation of the process in which T cells are inhibited by Tregs. High expression of TOP2A may cause high level of Treg infiltration, then CD8+ T-cells are inhibited by Tregs that results in the low level of CD8+ T-cell infiltration. On the other hand, CD8+ T-cells’ presence is often considered to have an association with favorable prognosis of tumors^44^. High TOP2A expression was statistically associated with poor prognosis for UCEC, so this result suggests the possibility that high TOP2A expression affects the patients’ outcomes via the inhibition of CD8+ T-cell infiltration. We also discovered that TOP2A expression in STAD was negatively associated with the infiltration level of B cells, CD8+ T-cells as well as DCs, which may be a factor in the development of STAD. As for neutrophils, whether they function as tumor-antagonizing or tumor-promoting factors depends on tumor types and developmental stages^44^. We found a positive correlation between neutrophils infiltration and TOP2A expression in the case of BLCA, BRCA, BRCA-Basal, COAD, KICH, KIRC, KIRP, LIHC, OV as well as PAAD, hinting the probability of neutrophils’ tumor-promoting role in above tumor types. Meanwhile, CAFs’ infiltration presented a statistically positive relationship with TOP2A expression in several tumor types, and this result manifested a potential role of CAFs in the tumor occurrence and promotion. Contrarily, we observed a negative relationship between CAFs infiltration and TOP2A expression in BRCA. It has been confirmed that CAFs play a stimulative role in Tregs’ infiltration, but high Tregs’ infiltration makes CAFs arrested at the G2/M phase^45^. Combining Tregs’ close interaction with CAFs and a positive association between TOP2A expression and Tregs’ infiltration in BRCA, we considered that high TOP2A expression may promote the arrest of CAFs by Tregs to affect CAFs’ growth in BRCA. High expression of TOP2A in BRCA may cause high level of Treg infiltration. Then high Tregs’ infiltration makes CAFs arrested at the G2/M phase to affect CAFs’ growth, and causes the low level of CAF infiltration. Additionally, M1 macrophages are viewed as antineoplastic factors, and M2 macrophages are regarded as tumor-promoting factors. The negative relationship between TOP2A expression and M1 macrophages’ infiltration level in TGCT and THYM suggests that high TOP2A expression may inhibit the function of M1 macrophages to promote the tumor progression. Besides, the positive association between the infiltration level of M2 macrophages and TOP2A expression in GBM hints a possibility that high TOP2A expression stimulates M2 macrophages’ tumor-promoting function in GBM.

As for antitumor therapy, TOP2A is viewed as a vital target, but the clinical efficiency of therapy targeting TOP2A may be limited by resistant tumor cells^46^. Combination therapy has been recommended to maximize drugs’ clinical effects. It has been reported that the combination therapy targeting CTLA-4 and PD-1 has great effects in the increase of median survival in many tumor types^47^. Additionally, the combination of TIGIT and PDCD1 blockade promotes CD8+ T-cell proliferation and function, and plays a significant role in the improvement of overall survival in preclinical trials^48,49^. To investigate the possibility of synergy between therapy targeting TOP2A and the inhibitors of immune checkpoints, our study analyzed the correlation between TOP2A and six immune checkpoints including CD274, CTLA-4, PD-1, PD-L1, TIM-3, LAG-3 and TIGIT. The positive association between TOP2A expression and the expression of above immune checkpoints hints that combination therapy targeting TOP2A and one of above immune checkpoints may have antitumor synergism and improve therapeutic efficacy. Thus, the interactions between TOP2A and immune checkpoints need to further investigate for more therapeutic choices and the improvement of response rates.

Taken together, our first pan-cancer analyses of TOP2A indicated its widespread over-expression in different cancer types. High expression of TOP2A was related to poor prognosis and advanced pathological stages in most cases, and TOP2A genetic alterations was existed in some tumor types. Furthermore, we built the upstream regulatory networks of TOP2A in KIRC, KIRP, LIHC and LUAD. TOP2A expression was generally positively correlated with cancer associated fibroblasts, M0 and M1 macrophages, and immune checkpoints. Our work of TOP2A pan-analysis contributes to understanding the prognostic and immunological roles and potential upstream molecular mechanism of TOP2A in different cancers.

## Supplementary Information

Supplementary Legends.

Supplementary Figure S1.

Supplementary Figure S2.

Supplementary Figure S3.

Supplementary Figure S4.

Supplementary Figure S5.

Supplementary Table S1.

Supplementary Information 8.

## Data Availability

Publicly available dataset Xena (https://xenabrowser.net/) was analyzed in this study. The ID numbers include: TCGA-BLCA.htseq_fpkm.tsv, TCGA-BRCA. htseq_fpkm.tsv, TCGA-CHOL.htseq_fpkm.tsv, TCGA-COAD.htseq_fpkm.tsv, TCGA-ESCA.htseq_fpkm.tsv, TCGA-GBM.htseq_fpkm.tsv, TCGA-HNSC. htseq_fpkm.tsv, TCGA-KICH.htseq_fpkm.tsv, TCGA-KIRC.htseq_fpkm.tsv, TCGA-KIRP.htseq_fpkm.tsv, TCGA-LIHC.htseq_fpkm.tsv, TCGA-LUAD. htseq_fpkm.tsv, TCGA-LUSC.htseq_fpkm.tsv, TCGA-PRAD.htseq_fpkm.tsv, TCGA-READ.htseq_fpkm.tsv, TCGA-STAD.htseq_fpkm.tsv, TCGA-THCA. htseq_fpkm.tsv, TCGA-UCEC.htseq_fpkm.tsv. The authors confirm that the data supporting the findings of this study are available within the article and its Supplementary materials.

## Abbreviations

TOP2A: Topoisomerase IIα
TCGA: The Cancer Genome Atlas
TMA: Tumor microenvironment
CAFs: Cancer associated fibroblasts
PD-L1: Programmed cell death ligand 1
CTLA-4: Cytotoxic T lymphocyte-associated antigen 4
TIM-3: T cell immunoglobulin domain and mucin domain 3
TIGIT: T cell immunoreceptor with Ig and ITIM domains
OS: Overall survival
DFS: Disease-free survival
PFS: Rogression-free survival
