# Supplementary material for: Oncogenic role and potential regulatory mechanism of topoisomerase IIα in a pan-cancer analysis
Source: Sci Rep. 2022 Jul 1;12:11161. doi: 10.1038/s41598-022-15205-7 (PMC9249858; doi:10.1038/s41598-022-15205-7)
Supplement: Supplementary file 1 — Supplementary Legends. [file 41598_2022_15205_MOESM1_ESM.docx]

**Supplementary Figure S1**

Correlation between TOP2A and immune infiltration level of B cells (A), CAFs (B) and neutrophils (C).

**Supplementary Figure S2**

Correlation between TOP2A and immune infiltration level of DCs (A) and macrophages (B).

**Supplementary Figure S3**

Correlation between TOP2A and infiltration of different types of macrophages. (A) M0 macrophages. (B) M1 macrophages. (C) Correlation M2 macrophages.

**Supplementary Figure S4**

Correlation between TOP2A and immune infiltration level of CD8+ T-cells (A), CD8+ T-cells (B) and Tregs (C).

**Supplementary Figure S5**

A schematic representation involving TOP2A alterations, associated signaling pathway regulating Platinum resistance as well as associated immune cells.
