# Supplementary figures and images for: Oncogenic role and potential regulatory mechanism of topoisomerase IIα in a pan-cancer analysis
Source: Sci Rep. 2022 Jul 1;12:11161. doi: 10.1038/s41598-022-15205-7 (PMC9249858; doi:10.1038/s41598-022-15205-7)

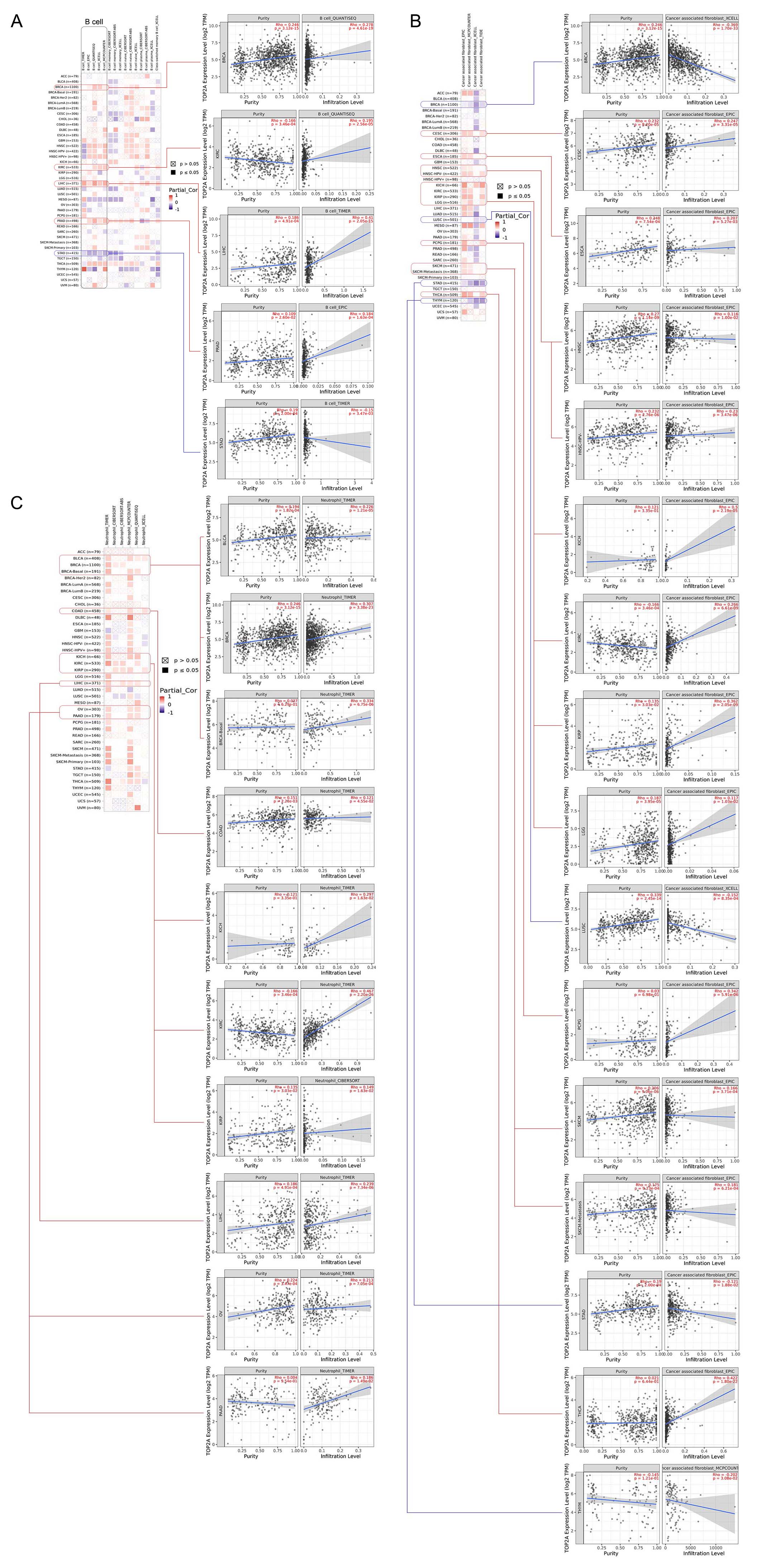

Supplement: Supplementary file 2 — Supplementary Figure S1. [file 41598_2022_15205_MOESM2_ESM.jpg]

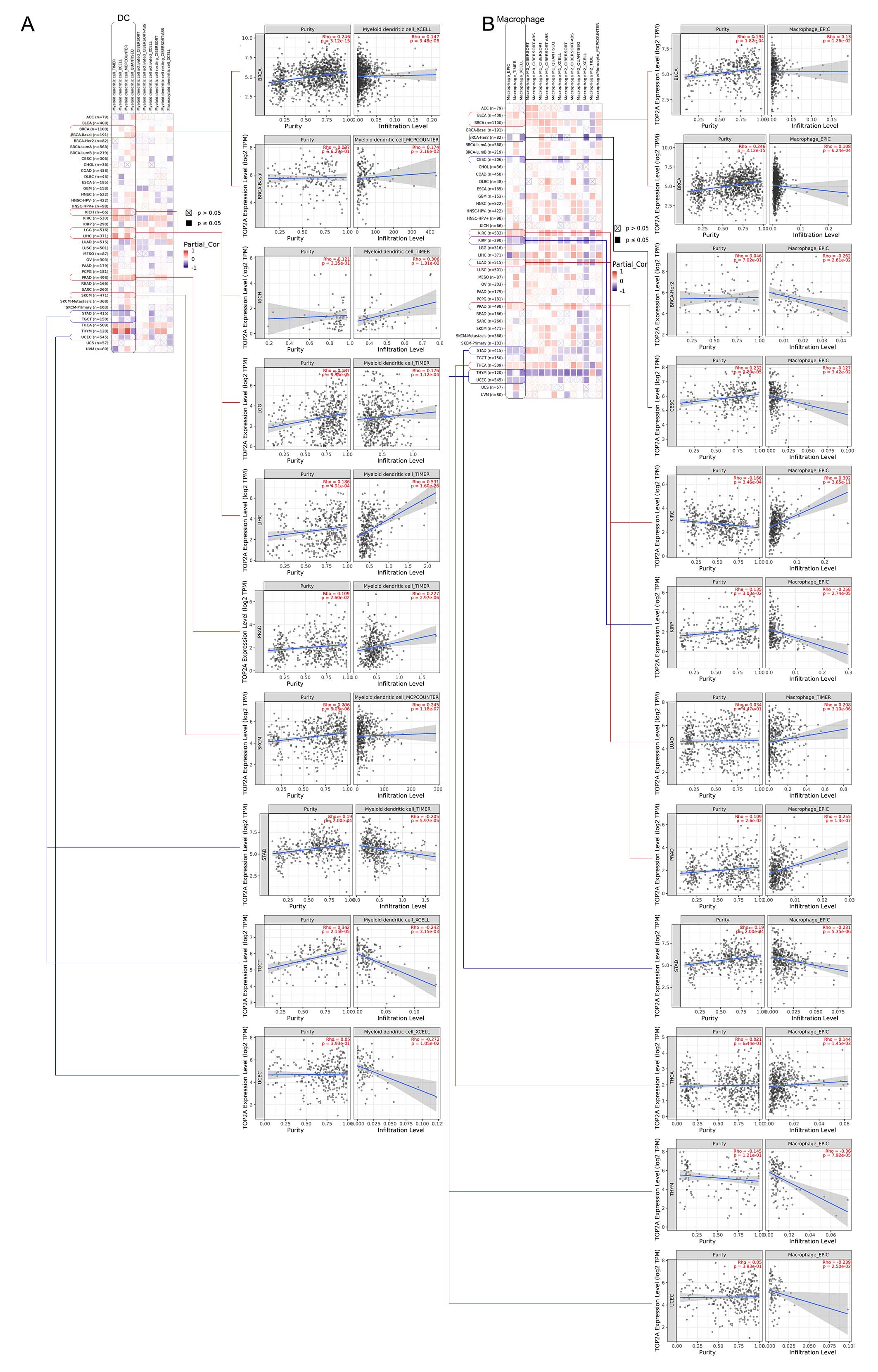

Supplement: Supplementary file 3 — Supplementary Figure S2. [file 41598_2022_15205_MOESM3_ESM.jpg]

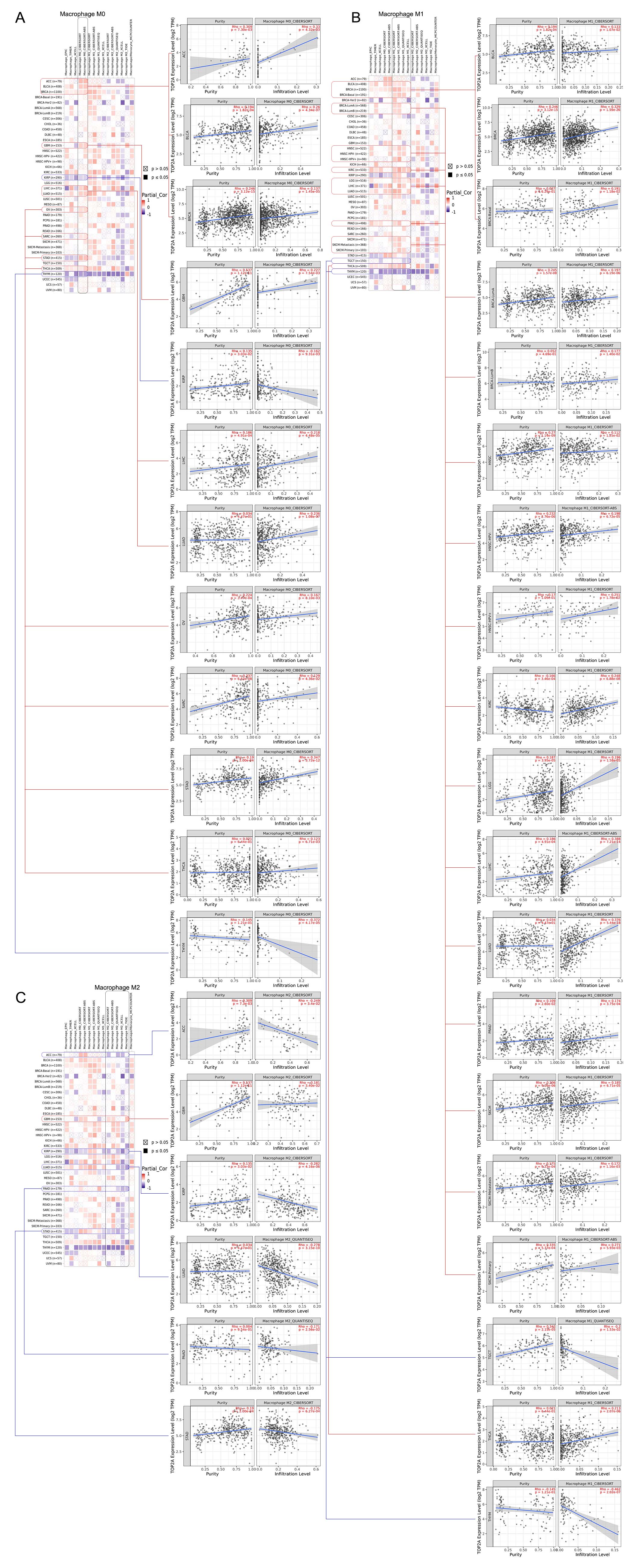

Supplement: Supplementary file 4 — Supplementary Figure S3. [file 41598_2022_15205_MOESM4_ESM.jpg]

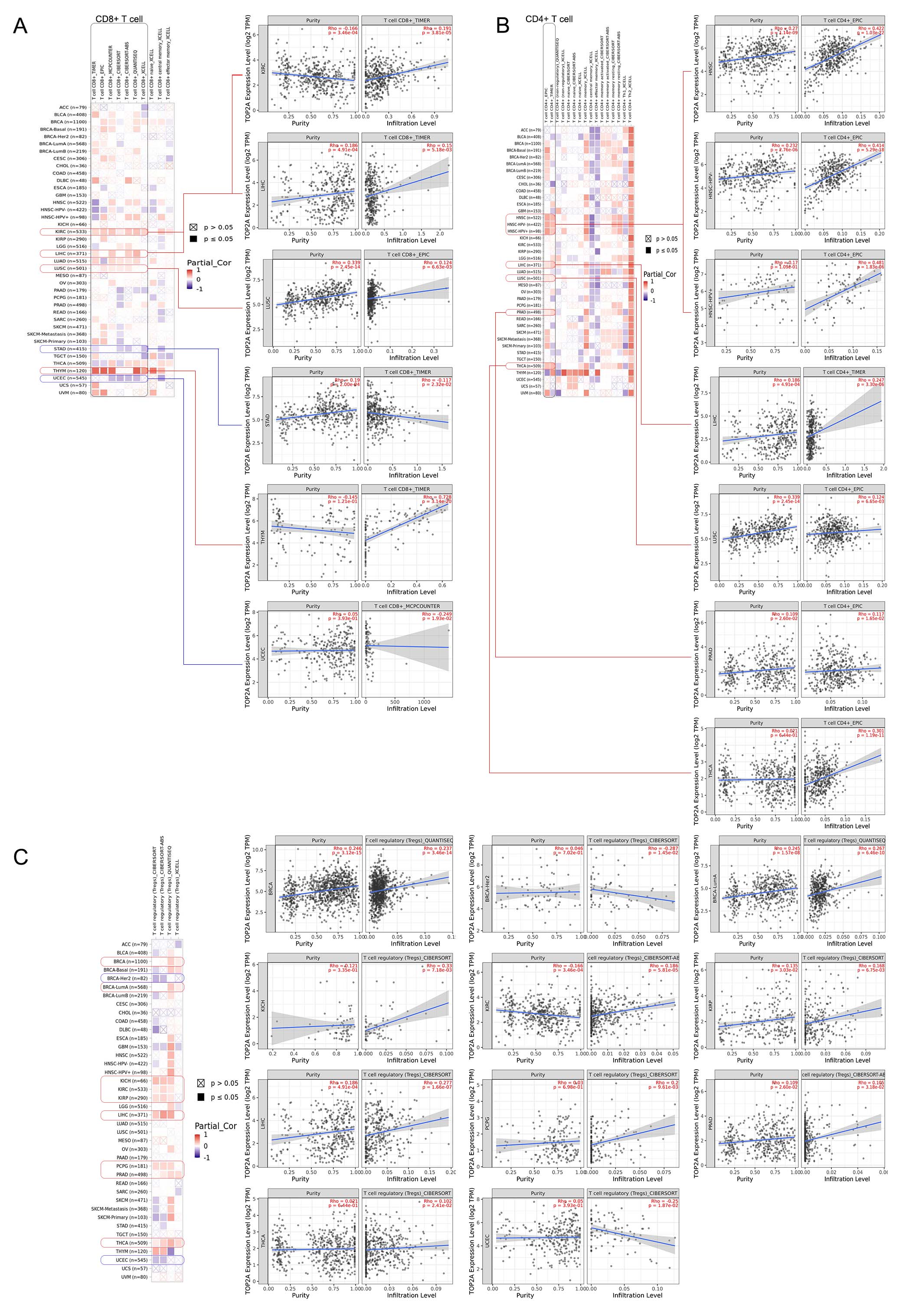

Supplement: Supplementary file 5 — Supplementary Figure S4. [file 41598_2022_15205_MOESM5_ESM.jpg]

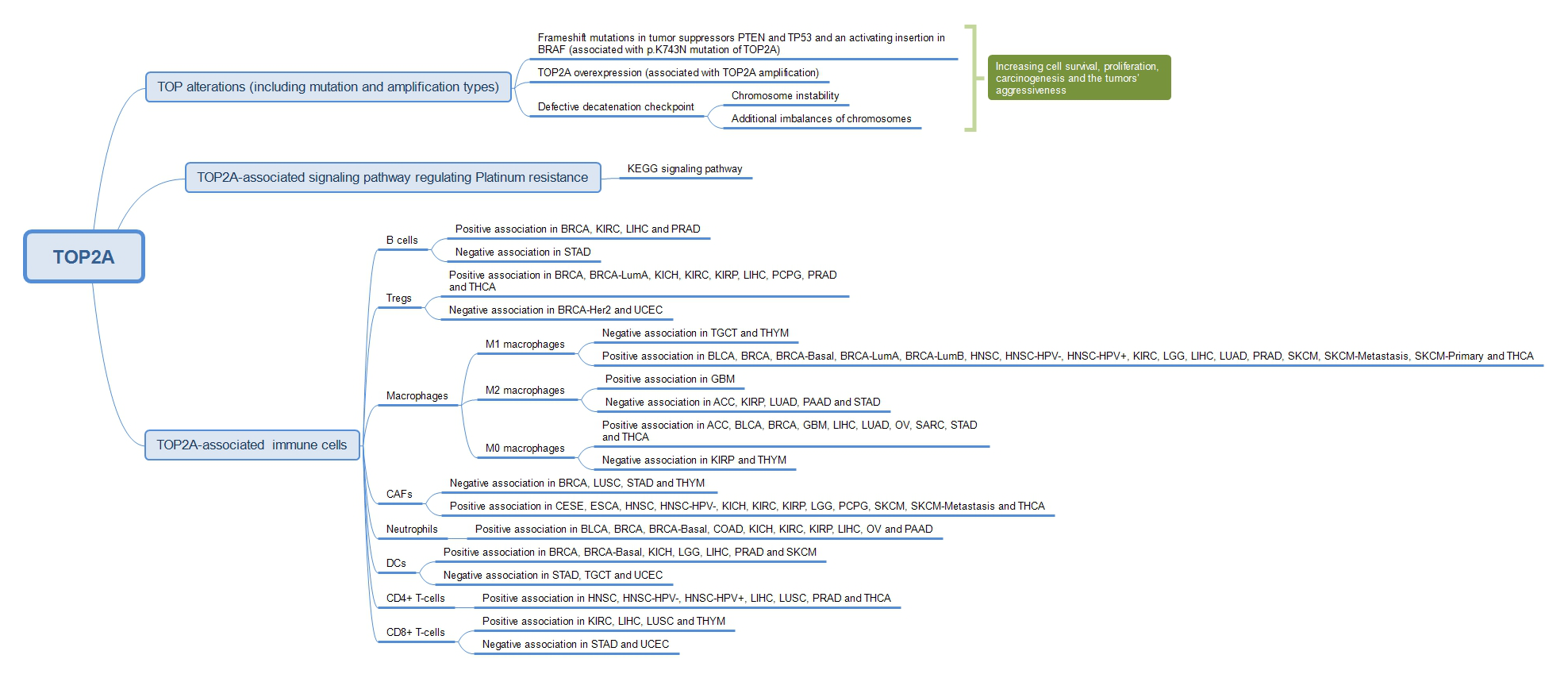

Supplement: Supplementary file 6 — Supplementary Figure S5. [file 41598_2022_15205_MOESM6_ESM.tiff]
