# Supplementary material for: Oncogenic role and potential regulatory mechanism of topoisomerase IIα in a pan-cancer analysis
Source: Sci Rep. 2022 Jul 1;12:11161. doi: 10.1038/s41598-022-15205-7 (PMC9249858; doi:10.1038/s41598-022-15205-7)
Supplement: Supplementary file 7 — Supplementary Table S1. [file 41598_2022_15205_MOESM7_ESM.docx]

**Supplementary Table S1 The predicting upstream miRNAs of TOP2A, and these miRNAs have been supported by CLIP-Seq experiment**

| hsa-miR-26a-5p | hsa-miR-323a-3p | hsa-miR-362-3p |
| --- | --- | --- |
| hsa-miR-26b-5p | hsa-miR-335-5p | hsa-miR-423-5p |
| hsa-miR-96-5p | hsa-miR-431-5p | hsa-miR-888-5p |
| hsa-miR-101-3p | hsa-miR-329-3p | hsa-miR-543 |
| hsa-miR-139-5p | hsa-miR-410-3p | hsa-miR-320b |
| hsa-miR-182-5p | hsa-miR-485-5p | hsa-miR-1271-5p |
| hsa-miR-224-5p | hsa-miR-495-3p | hsa-miR-1297 |
| hsa-miR-144-3p | hsa-miR-524-5p | hsa-miR-1270 |
| hsa-miR-320a | hsa-miR-520d-5p | hsa-miR-3681-5p |
| hsa-miR-365a-3p | hsa-miR-577 | hsa-miR-5687 |
| hsa-miR-376c-3p | hsa-miR-620 | hsa-miR-766-5p |
| hsa-miR-377-3p | hsa-miR-411-5p | hsa-miR-365b-3p |
| hsa-miR-383-5p | hsa-miR-28-3p |  |
